# Radiation-induced bronchiolitis obliterans organizing pneumonia (BOOP) syndrome in breast cancer patients is associated with age

**DOI:** 10.1186/s13014-015-0393-9

**Published:** 2015-04-26

**Authors:** Keiko Nemoto Murofushi, Masahiko Oguchi, Masahiko Gosho, Takuyo Kozuka, Hideyuki Sakurai

**Affiliations:** Department of Radiation Oncology, University of Tsukuba, Ibaraki, Japan; Department of Radiation Oncology, Cancer Institute Hospital, The Japanese Foundation for Cancer Research, Tokyo, Japan; Department of Clinical Trial and Clinical Epidemiology, Faculty of Medicine, University of Tsukuba, Tsukuba, Ibaraki Prefecture Japan

**Keywords:** Breast Cancer, Postoperative radiotherapy, Radiation-induced bronchiolitis obliterans organizing pneumonia syndrome, Breast-conserving therapy, Post-mastectomy radiation therapy

## Abstract

**Background:**

Radiation-induced bronchiolitis obliterans organizing pneumonia (BOOP) syndrome is a rarely observed phenomenon characterized by infiltration of the lungs outside of the radiation field, differentiating it from radiation pneumonitis (RP).The risk factors for radiation-induced BOOP (RT-BOOP) remain unclear and controversial. We retrospectively analyzed the incidence and risk factors for RT-BOOP associated with radiation therapy (RT) after breast conserving surgery (BCS) and post-mastectomy radiation therapy (PMRT).

**Methods and materials:**

We analyzed 1,176 breast cancer patients treated with RT after BCS or PMRT between March 2005 and September2008 at the cancer institute hospital of the Japanese foundation for cancer research. Chest radiographs were routinely obtained every three to six months for at least 12 months after surgery, as well as when the patients experienced respiratory symptoms or fever.

**Results:**

RT-BOOP syndrome was diagnosed in 16patients (1.4%), including12BCS patients (1.3%) and four PMRT patients (1.8%). An older age (≥52 years old) was significantly associated with the incidence of RT-BOOP syndrome in a univariate analysis (p =0.023). The type of treatment (BCS or PMRT) and irradiated lung volume at 20 Gy (V20) were not significantly associated with the incidence of RT-BOOP syndrome in the entire patient cohort. In the multivariate analysis, age and smoking were the significant factor associated with RT-induced BOOP syndrome (p =0.044 and 0.049, respectively).

**Conclusions:**

RT-BOOP syndrome was a rarity, and the incidence for BCT cases was similar to that for PMRT cases. The irradiated lung volume was not significantly associated with RT-BOOP syndrome. An older age can predict the incidence of RT-BOOP syndrome.

## Background

Radiation therapy (RT) plays an important role in the management of patients with breast cancer. The majority of patients with early breast cancer have been treated with breast-conserving treatment (BCT), consisting of wide excision and postoperative RT. Post-mastectomy radiation therapy (PMRT) is mainly applied for locally advanced breast cancer patients with T3 and T4 disease, or with four or more positive axillary lymph nodes. Recent meta-analyses demonstrated that RT after breast-conserving surgery (BCS) and PMRT provided absolute improvements in the local five-year recurrence rate and the survival rates after 10 years or more [1,2].

Bronchiolitis obliterans organizing pneumonia (BOOP) is recently recognized the disorder associated with “organizing pneumonia”. It is mostly idiopathic, and possibly caused by various etiological factors, including pulmonary infections, drugs, collagen vascular disease or RT. The symptoms of BOOP syndrome are cough, fever, dyspnea, malaise or some combination of these [3]. Radiation-induced BOOP (RT-BOOP) syndrome, which is commonly seen outside of the radiation fields, is particularly observed several months after RT. It is different from typical radiation pneumonitis (RP) that occurs within the RT field. Recent reports showed that the incidence of RT-BOOP syndrome in breast cancer patients after BCT was 1.8-2.9% [4-10]. However, the risk factors remain unclear and controversial. For RT-BOOP syndrome of patients treated with PMRT, the available data is very limited. We retrospectively analyzed 1,176 patients treated with BCT or PMRT, and evaluated the incidence and risk factors for RT-BOOP syndrome in the Cancer Institute Hospital of the Japanese Foundation for Cancer Research.

## Methods and materials

### Patient characteristics

Between March 2005 and September 2008, 1,215 breast cancer patients underwent RT after BCS or PMRT. We analyzed 1,176 patients who underwent both surgery and RT at our institution, had no prior RT to the thorax that would result in an overlap of RT fields and had undergone no cardiovascular, pulmonary or esophageal surgery within one year of the treatment. The median follow-up periods were 25.5 months (range, 8.1 to 53.4 months) for all patients, 26.1 months (range, 8.1to 53.4 months) for BCT cases and 23.7 months (range, 8.2 to 52.1 months) for PMRT cases. The characteristics of the 1,176 patients are shown in Table 1. All patients were females, and 19 patients were irradiated for bilateral breast cancer. The majority of the patients were in clinical stage I (n = 477) or II (n =415), therefore, 948 patients were classified into the BCT group, while 228 patients were in the PMRT group. This study was approved with the ethical review committee of department.

**Table 1:** **Patient characteristics (n = 1176)**

**Age(y), median (range)**	**52 (19-93)**
Breast site (right/left/bilateral)	584/573/19
Collagen vascular disease (yes/no)	2/1174
Smoking (yes/no/unknown)	201/973/2
Alcohol (yes/no/unknown)	439/735/2
Allergy (yes/no/unknown) Clinical stage (n = 1195 lesion)	400/775/1
0	163
I	477
IIA/IIB	270/145
IIIA/IIIB/IIIC	48/32/49
IV	2
ocult	4
Ipsilateral breast tumor recurrence	5
Histologictype (n = 1195lesion) Noninvasive ductal carcinoma	169
Invasive ductal carcinoma	929
Others	97

### Treatment strategy for breast cancer

**Figure 1:**
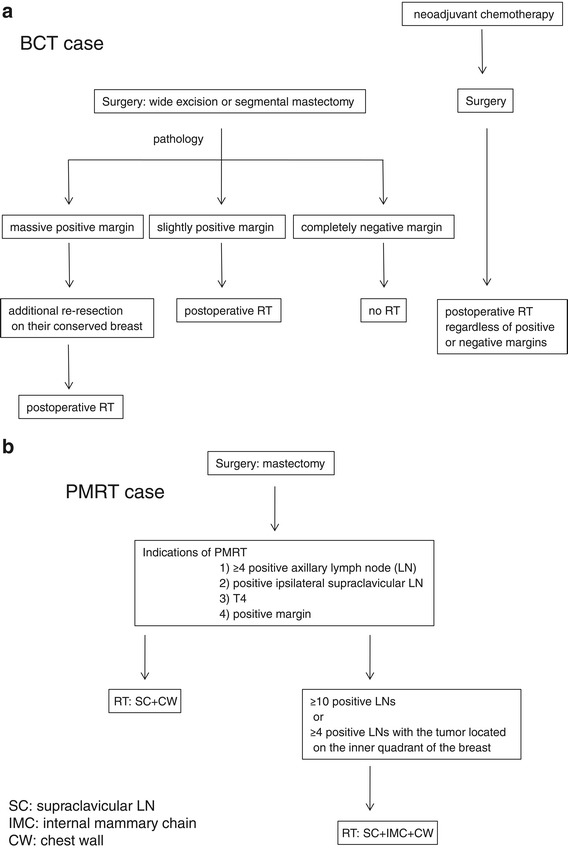
The treatment protocols for patients with breast cancer for breast-conserving therapy **(a)** and post-mastectomy radiation therapy **(b)**.

The treatment protocols for breast cancer in our institute are shown in Figure 1. In most cases of BCT (Figure 1a), wide excision was initially performed. The primary lesions were extracted by vertical resection from the skin to the fascia of the greater pectoral muscle. We have in-house clinical study of BCT without XRT for 20 years or more, and Nishimura reported the preliminary results that local recurrence of non RT-BCT patients with completely negative margin was 3.2% [11]. Patients with massive positive margins were treated with additional re-resection of the conserved breast, and all of those patients received RT regardless of the marginal status. In patients with T2N1 or more advanced stage disease were treated with neoadjuvant chemotherapy, and also received RT regardless of whether the margins were positive or negative. The entire breast was irradiated with 4 or 6 MV photon using opposing tangential fields. Electron beam boost was performed for most patients. Patients with positive margins or and the cases treated with neoadjuvant chemotherapy, but the patients who exhibited a pathological complete response to chemotherapy did not receive the electron beam boost. Cases with four or more positive lymph nodes underwent radiation treatments for both the entire breast and the supra/infraclavicular lymph node lesions.

The indications for PMRT (Figure1b) were: [1] four or more pathologically positive axillary lymph nodes, [2] positive ipsilateral supraclavicular lymph nodes, [3] T4 or [4] margin positive disease. The internal mammary chain (IMC) was irradiated if the patient had ten or more positive lymph nodes, or if four or more positive lymph nodes and the tumor were located on the inner part of the breast. An electron beam boost was performed for the cases at high risk of chest-wall recurrence, including those with positive margins and/or massive skin invasion, or the cases with unresected positive lymph nodes. The treatment fields were set up with simulators and/or a RT computed tomography planning system (Eclipse, Varian Medical Systems, PaloAlto, CA and Render-Plan, Elekta Oncology Systems, Inc, Norcross, GA). The lung volume irradiated at 20 Gy (V20) was calculated using dose-volume histogram.

The details of the radiotherapy are shown in Table 2. In the BCT group, the majority of patients (70.5%) received 60 or 66 Gy in 30 or 33 fractions of X-rays. Most patients (94.8%) received additional electron beam boost irradiation. On the other hand, the majority of PMRT patients (74.1%) were treated with 50 Gy in 25 fractions, and 74.6% of the patients did not receive boost irradiation. The median lung V20 was 6.2% (range, 0.2 to 31.8%) in the BCT group, whereas the median lung V20 was 13.7% (range, 2.3 to 24.7%) in the PMRT group.

**Table 2:** **Radiation therapy details**

	**BCT (n = 948)**	**PMRT (n = 228)**
Total dose and fractions^*^		
50 Gy in 25 fractions	42	169
50 Gy in 20 fractions	100	0
53.2 Gy in 20 fractions	114	0
60 Gy in 30 fractions	468	51
66 Gy in 33 fractions	200	4
70 Gy in 35 fractions	0	2
Others	43	2
Boost to the tumor bed (yes/no)^*^	917 (95%)/50 (5%)	58 (25%)/170 (75%)
Lung V20 (%), median (range)	6.2% (0.2-31.8)	13.7% (2.3-24.7)
Overall radiation therapy time (days), median (range)	42 days (21-65)	36 days (28-66)

^*^The 19 patients of 948 had bilateral breast cancers simultaneously and were treated with BCT, as a result, the treatment planning of 967 patients were evaluated.

The details of the pharmaceutical therapy are shown in Table 3. Endocrine therapy was administered to 67.7% of all patients. During radiation therapy, concurrent endocrine therapy was prescribed for most patients. Approximately half of the patients (51.6%) received neoadjuvant and/or adjuvant chemotherapy. No patients received chemotherapy concurrent with RT. Trastuzumab was administered to only 68 patients.

**Table 3:** **Drug therapy details (n = 1176)**

**Endocrine therapy (yes/no)**	**796 (68%)/380 (32%)**
Tamoxifen + LHRH agonist	106
Tamoxifen	289
Anastrozole	351
Letrozole	35
Exemestane	13
Others	2
Concurrent endocrine therapy (yes/no)	765 (96%)/31 (4%)
Chemotherapy (yes/no)	607 (52%)/569 (48%)
Preoperative	231
Postoperative	342
Pre and postoperative	32
unknown	2
Anthracycline + Taxiane-based regimen	471
Anthracycline-based regimen	119
Taxiane-based regimen	10
Others	7
Trastuzumab (yes/no)	68 (6%)/1108 (94%)

### Definition of radiation-induced BOOP syndrome

RT-BOOP syndrome was diagnosed based on the criteria proposed by Crestani et al. [12], which included: [1] a history of radiation therapy to the breast within the past 12 months, [2] general and/or respiratory symptoms lasting for at least two weeks, [3] radiographic lung infiltrates outside the radiation port, [4] no evidence of a specific cause. We have routinely followed the patients routinely at three, six and 12 months after radiation therapy. Chest X-rays were routinely taken every three to six months for at least 12 months after surgery. In addition when patients had developed consistent respiratory symptoms or fever, additional chest X-rays was examined. In the case with general and/or respiratory symptoms lasting two or more weeks and lung infiltrative findings were observed on chest X-rays, computed tomography (CT) scanning was performed.

### Statistical analyses

Results are presented as number (proportion) of patients for categorical variables. The incidence of RT-BOOP syndrome was analyzed by use of Fisher’s exact test as a univariate analysis. In addition, the incidence was assessed by using a multivariate logistic regression model to identify risk factors of RT-BOOT syndrome. The risk factors in the multivariate model were set based on the result of the univariate analysis. The factors with p < 0.15 in the univariate analysis for all patients or for the BCT cases were included in a multivariate model. In the univariate analysis, age was categorized in less than 52 years (the median) and 52 years or above. Lung V20 was also categorized in 5% intervals as 0-4.9, 5.0-9.9, 10.0-14.9, 15.0-19.9, 20.0-24.9, 25.0-29.9 and 30.0-34.9%. In the multivariate logistic regression analysis, the age variable was treated as a continuous variable. The statistical analyses were performed using the SPSS Base System software program (SPSS, Chicago, IL) and the SAS software version 9.4 (SAS Institute, Cary, NC). A value of p < 0.05 was considered to be statistically significant.

## Results

**Figure 2:**
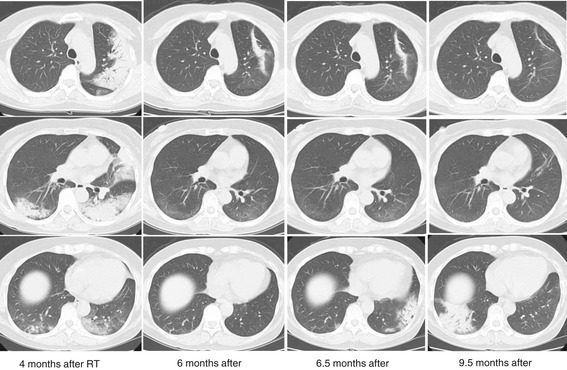
55-year-old patient received irradiation in the whole left breast (50 Gy/25 fractions) with an electron boost (10 Gy/5 fractions) to the partial breast. The lung V20 was 6.0%. Four months after RT following breast-conserving surgery, a severe cough, fever and decreased oxygen saturation measured by a pulseoximeter (SpO2; 88% at room air) were observed. Pulmonary lesions outside the irradiated field were observed on chest radiographs, which were located in the bilateral lungs on CT. Prednisolone was started at 30 mg/day based on a diagnosis of BOOP syndrome. The prednisolone was gradually tapered off, because the symptoms and pulmonary lesions were improved. However, the pulmonary lesions appeared again at 6.5 months after RT without any symptoms. Prednisolone was again administered until 7.5 months after RT. Although the pulmonary lesions appeared again at 9.5 months after RT, there were no symptoms.

**Figure 3:**
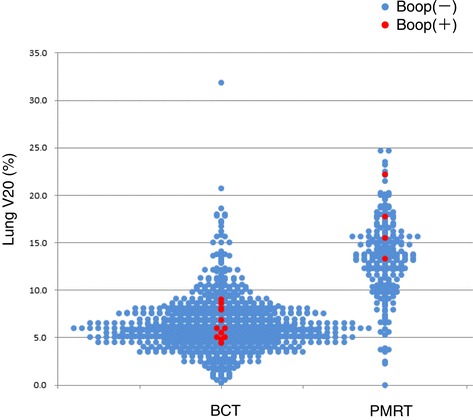
The lung V20 of all patients according to the type of treatment. The cases that developed radiation-induced BOOP are shown by red circles.

A typical case of RT-BOOP syndrome is illustrated in Figure 2. The details of clinical characteristics the patients with RT-BOOP syndrome are shown in Table 4. Sixteen patients (1.4%) of all developed RT-BOOP syndrome. Twelve of all (1.3%) were treated with BCT, and four of all (1.8%) were treated with PMRT. The median age of these 16 patients was 57 years (range, 43 to 79 years). Thirteen (81%) of them received endocrine therapy; tamoxifen (TAM) was administered to five patients, anastrozole (ANA) to seven and both TAM and a LHRH agonist were administered to one. Six (37%) of these patients received chemotherapy. The median lungV20 was 6.4% (range, 4.0 to 17.7%) for the 16 patients with RT-BOOP, 6.0% (range, 4.0 to 6.0%) for the twelve BCT cases and 14.5% (range, 4.8 to 17.7%) for the four PMRT cases. Figure 3 shows the actual V20 data for all of the patients. Although the V20 in the PMRT cases was higher than that in the BCT cases, no correlation was found between theV20 and incidence of RT-BOOP. On the other hand, eighteen patients (1.5%) of all developed grade 2 RP. Thirteen of them were treated with BCT (1.4%), and five of them were treated with PMRT (2.2%). No grade 3 or more RP was seen in all cases.

**Table 4:** **Details of the patients with BOOP syndrome (n = 16)**

**Patient no.**		**Endocrine therapy**	**CT**	**Lung V20(%)**	**Time after RT (mo)**	**Symptoms**	**Treatment**
1	BCT	TAM	-	7.9	7.6	cough	antitussive,
		LHRH-agonist					antibiotic
2	BCT	TAM	-	6.0	4.0	cough, pyrexia,	steroid,
						decreased SpO2	antibiotic
3	BCT	TAM	-	5.0	5.3	cough, pyrexia,	antitussive,
						fatigue	antibiotic
4	BCT	TAM	-	8.6	5.1	cough, pyrexia	antitussive,
							antibiotic
5	BCT	TAM	-	8.1	2.3	cough	antitussive,
							steroid
6	BCT	ANA	-	4.4	5.0	cough, fatuigue	antibiotic
7	BCT	ANA	-	6.0	4.7	cough	antitussive,
							antibiotic
8	BCT	ANA	-	5.1	3.0	cough, pyrexia	antitussive
9	BCT	ANA	-	9.0	4.5	cough	antitussive,
							antibiotic
10	BCT	ANA	-	4.8	3.1	cough, fatigue	antitussive,
							antibiotic
11	BCT	ANA	AC	6.8	8.6	cough	antitussive
			PAC				
12	BCT	-	CAF	5.6	6.0	cough	antibiotic
13	PMRT	TAM	CAF	17.7	3.3	cough, pyrexia	antitussive,
			PAC				antibiotic
14	PMRT	ANA	CAF	13.4	7.4	cough	antitussive,
			PAC				antibiotic
15	PMRT	-	CAF	15.6	3.9	cough, pyrexia	antibiotic
			PAC				
16	PMRT	-	CAF	22.0	5.3	cough, pyrexia,	antitussive
			PAC			fatigue	

*Abbreviations*

*CT* Chemotherapy.

*TAM* Tamoxifen.

*ANA* Anastrozole

*AC* Doxorubicin, Cyclophosphamide.

*PAC* Paclitaxel.

*CAF* Cyclophosphamide, Doxorubicin and Fluorouracil.

The median period from completion of RT to the diagnosis of RT-BOOP syndrome was 4.9 months (range, 2.3 to 8.6 months). The median period from the diagnosis of RT-BOOP syndrome to the last follow-up for all cases was 19.3 months (range, 3.9 to 39.9 months). The pulmonary findings of RT-BOOP disappeared in 14 of the patients during observation. The other two had been repeatedly relapsed BOOP syndrome over the years. Figure 2 shows the typical pulmonary findings of RT-BOOP marching both lungs on CT over 9 months. Steroids were administered to both of these patients, with durations of administration of seven days and 3.6 months.

Table 5 shows the factors associated with RT-BOOP syndrome for all patients, BCT cases, and PMRT cases as evaluated with a univariate analysis. For all patients, age was significantly associated with RT-BOOP syndrome (p = 0.023). There was no significant difference in the rate related to the use of endocrine therapy, but the incidence of RT-BOOP syndrome in the patients who received endocrine therapy (1.6%) was higher than the incidence of the patients without endocrine therapy (0.8%). The type of treatment (BCT or PMRT) and lungV20 were not significantly associated with the development of RT-BOOP syndrome. For the BCT cases evaluated with a univariate analysis, age was significantly associated with the incidence of RT-BOOP syndrome (p = 0.039). Smoking, chemotherapy and endocrine therapy were somewhat associated with RT-BOOP syndrome, though there was not statistically significant association (p = 0.10, 0.14 and 0.12, respectively). The lung V20 was not significantly associated with RT-BOOP syndrome. For the PMRT cases, none of the factors were significantly associated with RT-BOOP syndrome because the number of patients with RT-BOOP syndrome was too small and the statistical power was not enough to detect the association.

**Table 5:** **Univariate analysis of factors associated with RT-BOOT symdrome**

		**ALL (n = 1176)**			**BCT (n = 948)**			**PMRT (n = 228)**		
**Parameter**	**Level**	**n (%)**	**Incidence of BOOP syndrome, n (%)**	**p**	**n (%)**	**Incidence of BOOP syndrome, n (%)**	**p**	**n (%)**	**Incidence of BOOP syndrome, n (%)**	**p**
Age	<52y*	559 (48)	3 (0.5)	0.023	457 (48)	2 (0.4)	0.039	102 (45)	1 (1.0)	0.63
	>-52y	617 (52)	13 (2.1)		491 (52)	10 (2.0)		126 (55)	3 (2.4)	
OneSide	One side	1157 (98)	16 (1.4)	1.00	929 (98)	12 (1.3)	1.00	228 (100)	4 (1.8)	-
	Bilateral	19 (2)	0 (0.0)		19 (2)	0 (0.0)		0 (0)	0 (0)	
Allergy	Yes	400 (34)	6 (1.5)	0.79	328 (35)	5 (1.5)	0.76	72 (32)	1 (1.4)	1.00
	No	775 (66)	10 (1.3)		619 (65)	7 (1.1)		156 (68)	3 (1.9)	
	Unkonwn	1 (0)	0 (0.0)		1 (0)	0 (0.0)		0 (0)	0 (0.0)	
Smoking	Yes	201 (17)	5 (2.5)	0.17	147 (16)	4 (2.7)	0.10	54 (24)	1 (1.9)	1.00
	No	973 (83)	11 (1.1)		800 (84)	8 (1.0)		173 (76)	3 (1.7)	
	Unkonwn	2 (0)	0 (0.0)		1 (0)	0 (0.0)		1 (0)	0 (0.0)	
Chemotherapy	Yes	607 (52)	6 (1.0)	0.32	389 (41)	2 (0.5)	0.14	218 (96)	4 (1.8)	1.00
	No	569 (48)	10 (1.8)		559 (59)	10 (1.8)		10 (4)	0 (0.0)	
ET	Yes	796 (68)	13 (1.6)	0.29	635 (67)	11 (1.7)	0.12	161 (71)	2 (1.2)	0.58
	No	380 (32)	3 (0.8)		313 (33)	1 (0.3)		67 (29)	2 (3.0)	
RT	Yes	765 (96)	13 (1.7)	1.00	614 (97)	11 (1.8)	1.00	151 (94)	2 (1.3)	1.00
	No	31 (4)	0 (0.0)		21 (3)	0 (0.0)		10 (6)	0 (0.0)	
Trastuzumab	Yes	68 (6)	2 (2.9)	0.24	35 (4)	0 (0.0)	1.00	33 (14)	2 (6.1)	0.10
	No	1108 (94)	14 (1.3)		913 (96)	12 (1.3)		195 (86)	2 (1.0)	
Treatment	BCT	948 (81)	12 (1.3)	0.53	-	-	-	-	-	-
	PMRT	228 (19)	4 (1.8)							
Boost	Yes	963 (82)	13 (1.3)	1.00	902 (95)	12 (1.3)	1.00	61 (27)	1 (1.6)	1.00
	No	213 (18)	3 (1.4)		46 (5)	0 (0.0)		167 (73)	3 (1.8)	
Irradiation to the regional LNs	Yes	269 (23)	4 (1.5)	0.77	58 (6)	0 (0.0)	1.00	211 (93)	4 (1.9)	1.00
	No	907 (77)	12 (1.3)		890 (94)	12 (1.3)		17 (7)	0 (0.0)	
Lung V20	0-4.9%	280 (24)	2 (0.7)	0.19	274 (29)	2 (0.7)	0.62	6 (3)	0 (0.0)	0.32
	5.0-9.9%	620 (53)	10 (1.6)		586 (62)	10 (1.7)		34 (15)	0 (0.0)	
	10.0-14.9%	166 (14)	1 (0.6)		62 (7)	0 (0.0)		104 (46)	1 (1.0)	
	15.0-19.9%	93 (8)	2 (2.2)		22 (2)	0 (0.0)		71 (31)	2 (2.8)	
	20.0-24.9%	14 (1)	1 (7.1)		2 (0)	0 (0.0)		12 (5)	1 (8.3)	
	25.0-29.9%	0 (0)	0 (0.0)		0 (0)	0 (0.0)		0 (0)	0 (0.0)	
	30.0-34.9%	1 (0)	0 (0.0)		1 (0)	0 (0.0)		0 (0)	0 (0.0)	
	Unkonwn	2 (0)	0 (0.0)		1 (0)	0 (0.0)		1 (0)	0 (0.0)	

P: by Fisher’s exact test.

*: median of age.

*Abbreviations*

*BOOP* Bronchiolitis obliterans organizing pneumonia.

*ET* Endocrine therapy.

*RT* Radiation therapy.

*LNs* Lymph nodes.

*BCT* Breast conserving therapy.

*PMRT* Postmastectomy radiation therapy.

In the multivariate analysis, age associated with the incidence of RT-BOOP syndrome for all patients (p = 0.044), whereas age was a borderline significant risk factor for the incidence in BCT cases (p = 0.098) (Table 6). The incidence of RT-BOOP syndrome for smoking patients was higher than that for non-smoking patients (p = 0.049 for all patients, p = 0.033 for BCT cases). The incidence of RT-BOOT syndrome for the PMRT cases was not analyzed by using the multivariate logistic regression model because the number of patients with RT-BOOP syndrome was too small for the statistical analysis (n = 4).

**Table 6:** **Multivariate logistic regression analysis for RT-BOOP syndrome**

		**ALL**		**BCT**	
**Parameter**	**Level**	**OR (95% CI)**	**p**	**OR (95% CI)**	**p**
Age	10 y increased	1.58 (1.01, 2.48)	0.044	1.54 (0.92, 2.57)	0.098
Smoking	Yes/No	3.09 (1.01, 9.48)	0.049	4.12 (1.12, 15.11)	0.033
ET	Yes/No	2.09 (0.59, 7.44)	0.25	5.50 (0.70, 43.29)	0.11
Chemotherapy	Yes/No	0.56 (0.20, 1.60)	0.28	0.28 (0.06, 1.32)	0.11

*OR* odds ratio.

*CI* confidence interval.

## Discussion

The prognosis of breast cancer patients has improved over the past few decades [12]. RT plays an important role in the management of patients with breast cancer, and several studies showed a reduction of locoregional recurrence in breast cancer patients receiving postoperative irradiation [13]. In our previous study, the ipsilateral breast tumor recurrence rate after postoperative RT was only 1.6% [11]. Although the adverse events induced by postoperative RT for patients with breast cancer are generally mild, RT-BOOP syndrome does rarely occur, and can threaten the quality of life (QOL) for patients. It is necessary early identifying and treating the symptoms associated with RT-BOOP, such as cough, fever, dyspnea and/or fatigue and the QOL should be improved.

Radiographic lung infiltration of RP occurs inside the irradiated area of the lungs [14], and the percentage of the total lung volume exceeding 20 Gy (V20) can predict the development of Grade 2 or greater RP [15]. A recent study reported that radiation techniques delivering doses of 10 to 15 Gy to large lung volumes were significantly correlated with the incidence of RP [16]. RP is recognized as a direct lung injury caused by irradiation, while RT-BOOP syndrome is recognized as an indirect lung injury related to an autoimmune process [4,17]. We estimated an association between lung V20 and the development of the BOOP syndrome. V10 and V5 are not clearly different from V20 in treatment planning with opposing tangential fields. In this study we evaluate lung V20 among the irradiated lung parameters.

The incidence of RT-BOOP syndrome after BCT was reported to be 1.2 -2.9% (Table 7) [4-10]. However, it was unclear whether there was an association between the irradiated lung dose and volume and the incidence of RT-BOOP. Kubo et al. reported that the central lung distance (CLD) >1.8 cm, which was considered to be correlated with the irradiated lung volume, was significantly associated with RT-BOOP syndrome [10]. Katayama et al. reported that a CLD ≥ 3 cm was not significantly associated with RT-BOOP [7]. In our study, which investigated over 1,000 patients at a single institute, the lung V20 was not a significant factor predicting the development of RT-BOOP.

**Table 7:** **Reports of radiation induced BOOP syndrome**

**Authors**	**Year**	**Investigation**	**Materials**	**Number of patient**	**Incidence (%)**	**Related factor**
Takigawaetal [4]	2000	single institute	BCT	157	2.5	Immunologic reaction
Miwaetal [5]	2004	single institute	BCT	206	2.4	not evaluated
Ogoetal [6]	2008	multi-institute	BCT	2056	1.8	no factor was reported
Katayamaetal [7]	2009	multi-institute	BCT	702	2.3	age >50, concurrent
Kuboetal [8]	2009	multi-institute	BCT	413	2.9	endocrine therapy irradiated lung volume
Ogoetal [9]	2010	single institute	BCT	616	1.9	no factor was reported
Oieetal [10]	2013	single institute	BCT	428	1.2	radiation pneumonitis
Thisstudy	2014	single institute	BCT and PMRT	1176	1.4	older age

Some physicians have expressed concern about the potential for an increased incidence of RT-BOOP in PMRT group, who have a larger irradiated lung volume than BCT cases. In our study, the median lung V20 was 6.2% (0.2-31.8%) for BCT cases, while it was 13.7% (2.3-24.7%) for PMRT cases. However, the type of treatment (BCT/PMRT) was not significantly associated with the incidence of RT-BOOP syndrome (p = 0.53), even though the PMRT cases received radiation to a wider lung area than BCT cases. Additionally, several previous studies surveyed the incidence of RT-BOOP syndrome for BCT cases, but not for PMRT cases. In the present study, we also clarified that the incidence of RT-BOOP in PMRT cases, which was 1.8%, was similar to that in BCT cases. Based on our long-term follow up study, we concluded that the irradiated lung volume does not predict the development of RT-BOOP syndrome.

The patient age (≥52 years) was significantly associated with RT-BOOP syndrome for all 1,176 patients and for the BCT cases in the univariate analyses, however it was not a significant risk factor for PMRT cases in this study. In the multivariate analysis, age associated with the incidence of RT-BOOP syndrome for all patients (p = 0.044). The number of PMRT cases was smaller, it is necessary to analyze the larger number of PMRT cases for assessment whether age was a significant risk or not. But three of the four PMRT cases with RT-BOOP syndrome were more than 52 years old. We therefore conclude that age is the most important factor for predicting the development of RT-BOOP in breast cancer patients.

Endocrine therapy, including TAM and aromatase inhibitors, reduced the recurrence rate and improved the survival for the patients with endocrine-responsive breast cancer [18,19]. It has been reported that the concurrent use of TAM and RT increased the risk of grade 2 or greater subcutaneous fibrosis [20] and lung fibrosis [21]. Katayama [7] reported that concurrent endocrine therapy (ET) increased the risk of RT-BOOP syndrome. ET was not significantly associated with RT-BOOP syndrome in our study (p = 0.25). In our institution, since concurrent ET and RT were conducted for most of the patients, it is unknown whether the concurrent or sequential use of ET and RT has an impact on the incidence of RT-BOOP. However, there were no differences in the incidence between patients taking TAM and other hormone treatments, including aromatase inhibitors, in our study.

Chemotherapy was not significantly associated with RT-BOOP syndrome in this study (p = 0.28). 10 ofthe12 patients with RT-BOOP syndrome had not received chemotherapy. Since one of the suggested mechanisms underlying the RT-BOOP included hypersensitivity or an allergic response, it is possible that chemotherapy may reduce these immune responses. However, it is necessary to evaluate more cases to clarify the true correlation between chemotherapy and BOOP.

In this study, trastuzumab was not significantly associated with RT-BOOP. However, only 68 of the 1,176 patients were administered trastuzumab, so it will be necessary to analyze the correlation between trastuzumab and RT-BOOP in more patients receiving trastuzumab. The concurrent use of trastuzumab and RT for BCT patients was previously reported not to be associated with an increased rate of acute adverse events, including RP [22], and further follow-up will be necessary to evaluate the impact on late adverse events.

It has been reported that smoking depressed the frequency of RP in breast cancer and esophagus cancer patients [23]. Bjermer suggested that ongoing smoking during RT decreased an inflammatory response in the lung tissue induced by radiation [24]. The RT-BOOP syndrome is considered to have different mechanism from occurrence of RP, although immune response may play some role for both pulmonary reaction.

In this study, smoking was not significantly associated with RT-BOOP syndrome for all cases, BCT cases and PMRT cases in a univariate analysis (p = 0.17, 0.10 and 1.00, respectively). In the multivariate analysis, however, the incidence of RT-BOOP syndrome for smoking patients was higher than that for non-smoking patients (p = 0.049 for all patients, p = 0.033 for BCT cases). The association between RT-BOOP syndrome and smoking was not evaluated in the previous data. In this study, smoking group included several smoking habits including heavy and former smoker, however, very few current smoker during RT were included. In order to explain the association between smoking habit and RT-BOOP syndrome, we need further study using large data base.

In conclusion, the incidence of RT-BOOP syndrome was 1.4% for all 1,176 patients, and there were no significant differences between the BCT cases (1.3%) and for PMRT cases (1.8%). The lung V20 was not correlated with the development of RT-BOOP. The older age is the most important factor for the prediction of RT-BOOP syndrome in breast cancer patients who receive radiation therapy. We should pay attention to old breast cancer patients treated with postoperative RT to have the early diagnose of RT-BOOP syndrome.
